# Endovascular management of femoral access-site and access-related vascular complications following percutaneous coronary interventions (PCI)

**DOI:** 10.1371/journal.pone.0230535

**Published:** 2020-03-19

**Authors:** Nadjib Schahab, Refik Kavsur, Thorsten Mahn, Christian Schaefer, Alexander Kania, Rolf Fimmers, Georg Nickenig, Sebastian Zimmer

**Affiliations:** 1 Department of Internal Medicine II-Cardiology, Pulmonology and Angiology, University Hospital Bonn, Bonn, Germany; 2 Department of General, Visceral, Thoracic and Vascular Surgery, University Hospital Bonn, Bonn, Germany; 3 Institute of Medical Biometry, Informatics and Epidemiology, University Hospital Bonn, Bonn, Germany; Klinikum Region Hannover GmbH, GERMANY

## Abstract

**Background:**

Major vascular complications (VCs) of ilio–femoral arterial access after percutaneous coronary interventions are infrequent, but are associated with increased mortality and morbidity. Routine endovascular repair of VCs is becoming the treatment of choice, especially for patients who cannot tolerate vascular surgery due to advanced cardiovascular disease or are in a bailout situation. Here, we review the different types of vascular access site complications associated with percutaneous coronary interventions (PCIs) and assess the safety and efficacy of endovascular treatment.

**Methods:**

Data were retrospectively analysed from patients who experienced VCs after transfemoral PCIs, from December 2014 to May 2018. During this period, out of 2833 patients who underwent femoral coronary interventions, 53 (1.9%) experienced major VCs.

**Results:**

In total, 40/53 (75.5%) cases with major VCs led to unplanned endovascular repair and 13/53 (24.5%) cases required surgical repair. VCs included 17 (32.1%) retroperitoneal bleeding events (BARC-2, 3a,b), 20 (37.7%) intimal dissections, and 16 (30.2%) femoral pseudoaneurysms. Overall, 32 (60.4%) patients received a covered stent, two (3.8%) received a nitinol stent, five (9.4%) patients with dissections were treated with prolonged balloon angioplasty alone, and one patient with femoral pseudoaneurysm underwent thrombin injection with simultaneous balloon occlusion. The mean hospital stay for patients after endovascular treatment was 11.06 ± 5.2 days, while for patients after surgical repair it was 17 ± 8.2 days. Endovascularly treated patients were transfused with red blood cells (13/40 32.5% vs. 2/13 15.4%) significantly more often than patients treated surgically, although surgically treated patients received more red blood cell concentrates per unit than endovascularly treated patients (1 ± 0.47 vs. 2 ± 0.93). During the one-year follow-up, no intermittent claudication was reported, and no patient required secondary endovascular or surgical repair.

**Conclusions:**

For patients who cannot tolerate vascular surgery due to advanced cardiovascular disease or are in a bailout situation, endovascular management of VCs following PCIs seems to be a feasible and safe treatment option, and represents an alternative to surgical repair in life-threatening situations. Endovascular treatment was associated with significantly fewer red blood cell concentrates per patient and fewer days in hospital than surgical treatment.

## Introduction

Transradial artery access for percutaneous coronary angiography represents the primary access modality and is strongly recommended, although femoral vascular access is necessary for numerous procedures, especially for more complex coronary interventions requiring large-bore access. In previous analyses, the total vascular complication rates (bleeding and other vascular complications) after femoral puncture for percutaneous interventions ranged from 2% to 7.9% [1]. Vascular complications (VCs) associated with femoral arterial access after percutaneous coronary interventions (PCIs) [2] remain an important source of morbidity and mortality and are correlated with longer hospital stays, greater nursing requirements, and increased in-hospital and long-term rehabilitation costs [3]. Surgical repair is an effective treatment with a high success rate for iatrogenic femoral access lesions. However, it also has its own associated risks, such as wound complications, intraoperative myocardial infarctions, and prolonged hospital stay [4]. Furthermore, patients undergoing PCI often have significant comorbidities that increase intra- and post-operative morbidity and mortality. Endovascular treatment represents an alternative to surgical repair in bailout situations. The most frequent and major complications include uncontrollable groin and/or retroperitoneal bleeding, arterial dissection with or without associated thrombosis, and pseudoaneurysm (PA) with and without arteriovenous fistula (AVF) [5–7]. In cases of VCs, early recognition and accurate intervention are essential for optimal management of patients.

The aim of this study was to assess the safety and efficacy of endovascular treatment for PCI-associated VCs at the femoral access site, focusing on the relevance of early diagnosis and prompt treatment using endovascular techniques.

## Materials and methods

### Study population

Data were retrospectively reviewed for patients with VCs after or during transfemoral PCI and were treated using either endovascular or surgical strategies from December 2014 to May 2018 at the Department of Cardiology and Angiology of University Hospital Bonn in Germany. All patients were screened in the clinical database (Orbis, AGFA Healthcare) of our department.

### Inclusion and exclusion criteria

All patients who underwent PCI received routine clinical examinations and were evaluated for typical signs of arterial injury. When VCs were suspected, Doppler ultrasonography (DUS) and/or computed tomography angiography were performed, based on the patient’s clinical status and accessibility of the suspected injured vessels with imaging techniques. Angiography was used to confirm diagnoses for all cases. Management of major vascular complications in patients with retroperitoneal bleeding and dissections with thrombosis or expanding PAs occurred during or immediately post intervention (within minutes) in the catheter laboratory, because of their clinical situation. Patients with PAs and dissection without thrombosis and in stable situations were diagnosed within a delayed interval during pre-discharge visits and routine ultrasound control.

The decision criteria to initiate interventional vascular repair were: urgency and limited time for surgical repair, due to unstable, life-threatening clinical status, and/or failure of conservative therapy upon review by the vascular team, consisting of a vascular surgeon, an interventional angiologist and a cardiologist. The criteria for stenting a PA after failed manual compression was an infected groin with extended haematoma, and patient’s choice for endovascular rather than surgical treatment. VCs located at the femoral bifurcation and rapidly expanding PAs in young patients with wide necks (> 3 mm) were treated surgically.

### Primary outcomes and follow-up data

Primary successful treatment was defined as a complete resolution of the VC without further therapy (i.e., conservative or surgical). Follow-up data were available from post-procedural, in-hospital visits at our clinic and when duplex ultrasound (DUS) was performed. Quantitative criteria to diagnose stenoses were peak systolic velocity and peak systolic velocity ratios and DUS within or beyond the stenosis compared with the adjacent proximal arterial segment, the presence or absence of turbulence, and the preservation of pulsatility. A peak systolic velocity ratio greater than 2 indicated stenosis greater than 50%.

Successful secondary treatment was defined as the absence of intermittent claudication and of the need for secondary endovascular or surgical therapy for the VC for 12 months following primary intervention.

### Statistical analyses

Data were retrospectively analysed using Microsoft Excel for Office 365^®^ (Redmond, WA, USA) and IBM^®^ SPSS^®^ software, version 26.0 (IBM Corporation, Armonk, NY, USA). Metric variables were described as mean ± standard deviation and compared with a t-test in normally distributed variables. For categorical variables, we used cross tables to present the absolute and relative frequency and verified independency with the Chi-Square Test. The level of statistical significance was set at p < 0.05.

### Ethical considerations

All data were fully anonymised before access and analysis. Ethics approval for the retrospective study was obtained from the local ethics committee of the University of Bonn. Interventions were conducted only after patients had provided informed consent for the procedure and for any and all relevant, medically appropriate care that should be provided in emergencies.

## Results

### Baseline characteristics

Baseline characteristics of patients according to treatment are summarised in **Table 1.** A total of 9833 PCIs (7000 transradial and 2833 transfemoral) were performed in our centre during the study period of 42 months. A total of 53 (1.9%) patients had major VCs that required treatment, with an estimated incidence of 1.9% for the study period. A total of 347 (12.2%) patients had minor complications (superficial haematomas [BARC-0, 1], PA and haemodynamically irrelevant peripheral arterial dissection) without any requirement for surgical or endovascular treatment or manual compression in case of PA; these cases were not further analysed.

**Table 1:** Baseline characteristics for all patients with major vascular complications.

	All major VCs	Endovascular	Surgical	p-value
	(n = 53)	(n = 40 [75.5%])	(n = 13 [24.5%])	
Age (years)	73.17 ± 11.8	72.05 ± 12.1	76.62 ± 10.21	0.611
Females, n (%)	28 (52.8)	22 (55.0)	6 (46.2)	0.751
Arterial hypertension, n (%)	41 (78.8)	30 (76,9)	11 (84.6)	0.709
Body mass index (kg/m^2^)	26.7 ± 6.0	26.9 ± 6.7	25.8 ± 3.2	0.050
Diabetes mellitus, n (%)	14 (26.9)	11 (28.2)	3 (23.1)	1.0
Smoking, n (%)	28 (54.9)	21 (53.8)	7 (58.3)	1.0
CAD, n (%)	43 (82.7)	33 (82.5)	10 (83.3)	1.0
STEMI, n (%)	23 (43.4)	16 (40.0)	7 (53.8)	0.522
AFib, n (%)	18 (34.0)	16 (40.0)	2 (15.4)	0.177
PAD, n (%)	24 (45.3)	16 (40.0)	8 (61.5)	0.212
COPD, n (%)	6 (11.3)	6 (15.0)	0 (0.0)	0.317
Anticoagulation*, n (%)	28 (56)	24 (60.0)	4 (40.0)	0.302

Values show frequencies and percentages or mean ± SD. *VCs* vascular complications; *CAD* coronary artery disease; *STEMI* ST-elevation myocardial infarction; *AFib* atrial fibrillation; *PAD* peripheral arterial disease; *COPD* chronic obstructive pulmonary disease

*Anticoagulation at index time.

The incidence of PA was 76/2833 (incidence 2.7%); 60/76 (78.9%) PAs were successfully treated with ultrasound-guided manual compression (38/76) and percutaneous thrombin injection (22/76).

In total, 17/53 (32.1%) patients experienced active retroperitoneal bleeding (BARC-2, 3a,b), 16/53 (30.2%) femoral PAs were noted after failed manual or ultrasound-guided compression (five [9.4%] with a concomitant AVF), and 20/53 (37.7%) patients underwent dissections, including 15/53 (28.3%) patients with vessel thrombosis. Depending on patient’s clinical status, endovascular treatment was initiated either immediately or within a specific interval after initial PCI (median 1.8 ± 2.5 days after the initial PCI). Among all major VCs, 13/53 (24.5%) patients required surgical repair and 40/53 (75.5%) patients received endovascular treatment (Table 2).

**Table 2:** Vascular complications and procedural characteristics.

	All patients (n = 53)	Endovascular	Surgical	p-value
		(n = 40 [75.5%])	(n = 13 [24.5%])	
**Major vascular complications**	** **			
Access sheath > 6Fr, n (%)	25(47.2)	21 (52.5)	4 (30.8)	0.574
Access sheath ≤ 6Fr, n (%)	23 (43.4)	20 (50)	3 (23.1)	0.500
Retroperitoneal bleeding, n (%)	17 (32.1)	15 (37.5)	2 (15.4)	0.183
Pseudoaneurysm, n (%)	16 (30.2)	13 (33.3)	3 (23.1)	0.730
Pseudoaneurysm with AV, n (%)	5/16 (9.4)	5 (12.5)	0 (0)	0.317
Dissection without thrombosis, n (%)	5 (9.4)	4 (10.0)	1 (7.7)	1.0
Dissection with thrombosis, n (%)	15 (28.3)	8 (20.0)	7 (53.8)	0.032
**Procedures and outcome**				
Ballon angioplasty, n (%)	5 (9.4)	5(12.5)		
Stenting (nitinol), n (%)	2 (3.8)	2 (5.0)		
Stenting (covered), n (%)	32 (60.4)	32 (80.0)		
Stent diameter, (mm)		5.53 ± 3.4		
Stent length, (mm)		28.52 ± 8.78		
Thrombin injection, n (%)	1 (1.9)	1 (2,5)		
Open surgical repair with thrombectomy	8 (15,1)		8 (61,5)	
Open surgical repair with patch angioplasty	5 (9.4)		5 (38.4)	
Wound infection, n (%)	4 (7.5)	0 (0.0)	4 (30.8)	0.002
Lymph fistula, n (%)	1(7.1)	0 (0.0)	1 (7.7)	1.0
Red blood cell concentrate, n (%)	15 (28.3)	13 (32.5)	2(15.4)	0.035
Red blood cell concentrate, units per patient	18 ± 0.62	1 ± 0.47	2 ± 0.93	0.002
Hospital stay (in days)	12,91 ± 6.7	11.06 ± 5.2	17.62 ± 8.2	0.003
Death	6 (11.3)	6 (15)	0	0.317

Values show frequencies and percentages, mean ± SD, *AV* arteriovenous fistula

The 40 patients in the endovascular treatment arm underwent the following treatments: thirty-two (60.4%) patients received covered stents (LIFESTREAM, Bard Peripheral Vascular, Tempe, AZ, USA and Fluency stent graft, C.R. BARD Inc, Murray Hill, NJ, USA), two (3.8%) were treated with nitinol self-expanding stents (Medtronic/ Protégé™ GPS™, Plymouth, MN, USA), and five (9.4%) patients with dissections were treated with prolonged balloon inflation alone. The mean stent size was 5.5 ± 3.4 mm x 28.5 ± 8.7 mm. One patient with a femoral pseudoaneurysm received thrombin injection and balloon occlusion simultaneously. Patients in the surgical group (n = 13) were treated either with surgical thrombectomy (n = 8) (one case without thrombosis and seven cases with thrombosis), or surgical repair using polypropylene suture. Arterial lacerations that were not suitable for primary repair were treated with graft or patch angioplasty in PA (n = 3) and retroperitoneal bleeding (n = 2) (Table 2).

The distribution of treatment modalities (endovascular vs. surgical treatment) according to complication groups was as follows: retroperitoneal bleeding (15/17 vs. 2/17), PA (13/16 [12 covered Stent, one patient received simultaneously thrombin injection and balloon occlusion] vs. 3/16), and dissection group (12/20 vs. 8/20).

In all endovascularly-treated VCs, either the transbrachial artery approach or the crossover technique was performed with a 6–8 Fr (8 Fr was needed in stent diameter > 8 mm) introducer sheath placed at the level of the ipsilateral internal iliac artery over a stiff guidewire. Heparin (2500–5000 IU) was injected intra-arterially. In cases with intimal dissections, a balloon catheter was first inflated at the target lesion site. Depending on the success of intimal adhesion and the target vessel size, in some cases, implantation of a stent graft was not necessary. Stent grafts with a diameter 1 mm larger than the vessel diameter were deployed at the target lesion/leaking point, with adequate proximal and distal landing zones. Angiography was performed consecutively to confirm that there was no remaining occlusion/extravasation and that the patency of the femoral artery was maintained. Endovascular treatment was selected in 15/17 (88%) cases of retroperitoneal bleeding. All endovascularly treated retroperitoneal bleedings were located in the distal of external iliac artery above the inguinal ligament. Only two (11.8%) cases of retroperitoneal bleeding, located at the femoral bifurcation, were treated surgically. Overall, 61.5% of patients in the surgical group had a dissection with occlusion and thrombosis and were treated with thrombectomy.

Primary success was achieved in all patients with complete resolution of the VC and no further therapy was required. Follow-up duplex scans were performed within 10 to 28 hours. No recurrence or failed repair events were detected. In addition, 12-month follow-up data were available for 40/53 (75.5%) patients (with a mean follow-up of 15 ± 13 months). In total, six (11.3%) patients in the endovascular group died during this time period (three due to infection unrelated to the procedure, two due to myocardial infarction, and one due to liver cirrhosis). No deaths occurred during the follow-up period in the surgically treated patients. For the remaining patients (47/53), no intermittent claudication was reported, and secondary endovascular or primary surgical treatment was not required. A three-month angiological follow-up via duplex sonography was performed in 50/53 patients. No significant (> 50%) stent graft stenosis or fracture was detected in ultrasound examination in these patients. In the surgical arm (n = 13), four (30.8%) cases developed wound infection, which in one (7.5%) case led to impaired postoperative wound healing that resulted from a lymph fistula in the groin with prolonged hospital stay (Table 2).

A significant difference in the length of hospital stay between the two groups was observed. The mean hospital stay was 11.06 ± 5.2 days for patients after endovascular treatment and 17 ± 8.2 days (p = 0.003) for patients (n = 13) after surgical repair. Endovascularly treated patients were transfused with red blood cells (13/40 32.5.1% vs. 2/13 15.4%) significantly more often than patients treated surgically, although surgically treated patients received more red blood concentrates per unit than endovascularly treated patients (1 ± 0.47 vs. 2 ± 0.93; p = 0.002) (Table 2).

## Discussion

Effective and rapid treatment is crucial for ilio–femoral access-related VCs following PCI. Here, we have shown that the application of endovascular strategies in these patients in a bailout situation results in safe and immediate repair of the VC with a 100% success rate, leading to a quick return to ambulatory activity and bypassing, in most cases, vascular surgery and its associated morbidity and mortality. One-year follow-up data indicated a good midterm outcome with no reported intermittent claudication and no need for secondary interventional and/or surgical treatment.

Compared with vascular surgery, immediate endovascular treatment in the catheterization lab bypasses transportation to the operating room and general anaesthesia, thus saving crucial time, particularly in retroperitoneal bleeding cases. Additionally, this technique prevents exposure to the risks of general anaesthesia in a group of patients who are often affected by advanced cardiovascular disease and reduces the length of hospital stay.

### Haemorrhage

**Fig 1:**
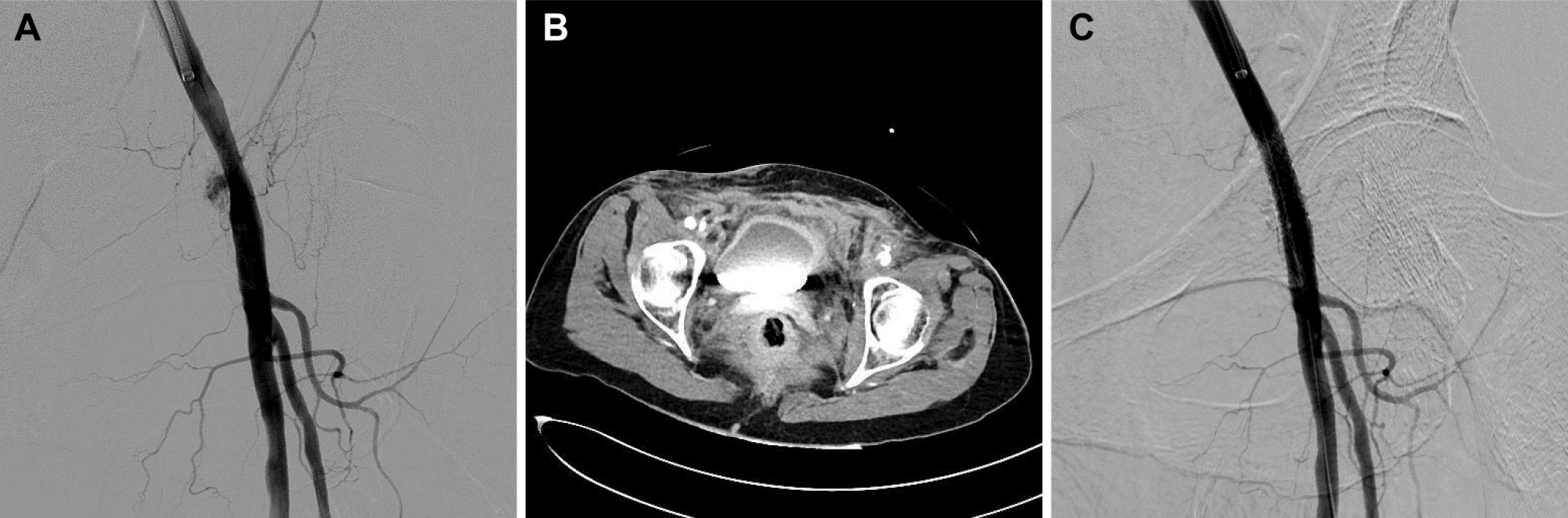
A 66-year-old female experienced a high retrograde puncture above the inguinal ligament, 2.5 cm cranial to the femoral head. Despite manual compression, a rapidly increasing groin haematoma developed. (a) Angiography demonstrated active extravasation from the iliac external artery, immediately above the level of the inguinal ligament. (b) Contrast-enhanced computed tomography scans demonstrated active extravasation from the external iliac artery and an accompanying haematoma. (c) Successful sealing of the perforation was achieved by deployment of an 8 x 26 mm covered stent (LIFESTREAM; C. R. Bard, USA) via a contralateral crossover approach.

Small superficial haematomas (BARC-0, 1) resolve spontaneously with time and only conservative treatment measures are required, such as the application of local pressure to the access site, prolonged bed rest, modification or interruption of anticoagulant and antiplatelet medication, keeping the patient well hydrated, and serial monitoring of blood cell counts. Retroperitoneal haemorrhage is the most severe form of access site bleeding, which is clinically associated with increased mortality [8]. In a recent study, Sedaghat et al. showed that implantation of a self-expanding nitinol stent graft in patients with vascular injuries (mainly bleeding) following transcatheter aortic valve replacement (TAVI) was a feasible and safe treatment and was associated with favourable short- and mid-term clinical outcomes [9,10]. In our patients with retroperitoneal bleeding, we experienced similar primary success with stenting in VCs after PCI, which is a less frequent event, probably due to the smaller access sheath diameters (Fig 1). We primarily used balloon mount covered stents (LIFESTREAM) in all patients with haemorrhages located in the external iliac artery and flexible, self-expending vascular prosthesis (FLUENCY) in the distal external iliac and proximal common femoral artery. Only two cases required surgical treatment, due to the VC being located at the femoral bifurcation and, thus, unsuitable for stenting.

### Femoral artery PA

**Fig 2:**
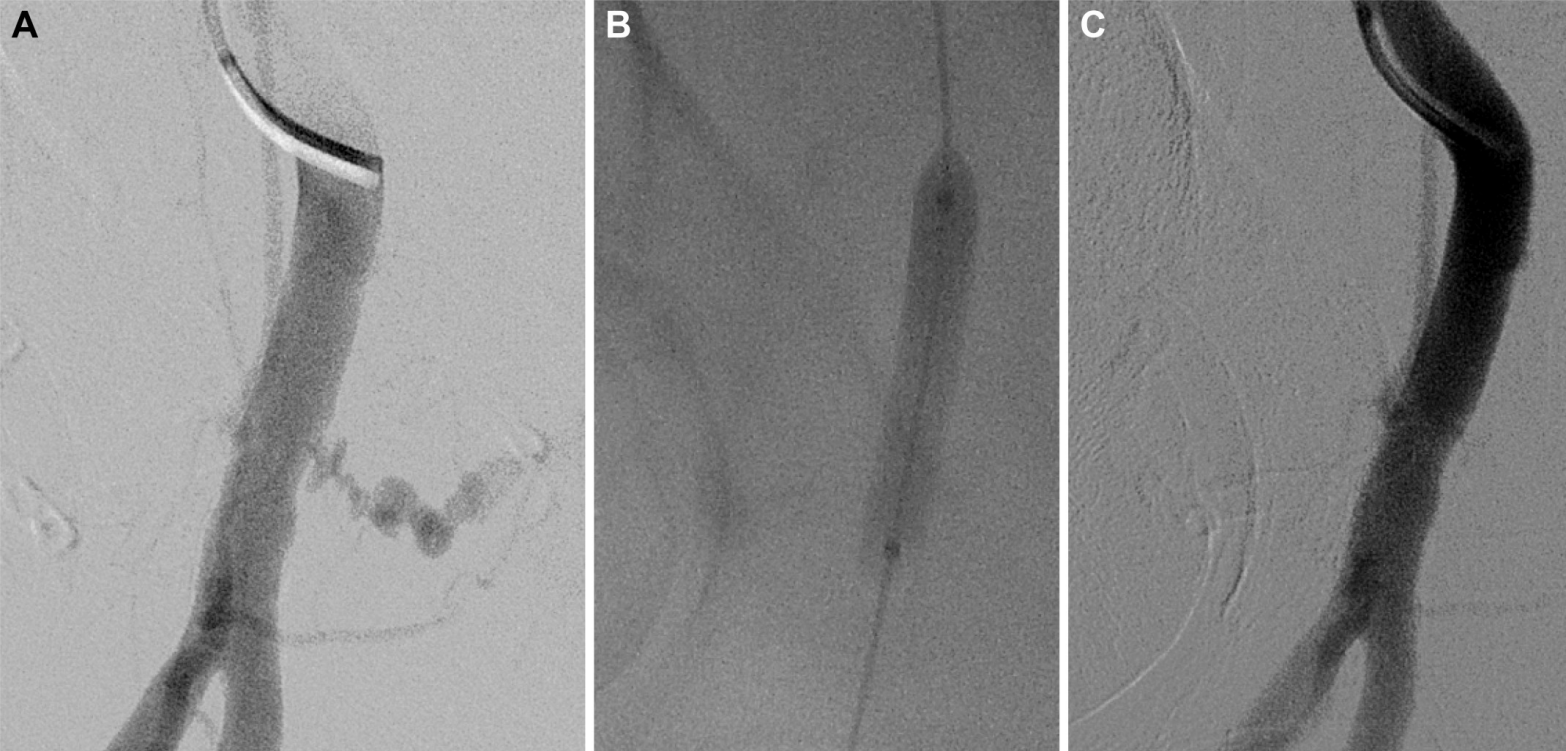
(a) Iatrogenic pseudoaneurysm after failed manual compression. (b) After placing an angioplasty balloon (6 mm in diameter) from a contralateral femoral approach and inflation, thrombin injection was performed under ultrasound guidance. (c) Angiography showing successful thrombosis of the pseudoaneurysm after percutaneous thrombin injection.

**Fig 3:**
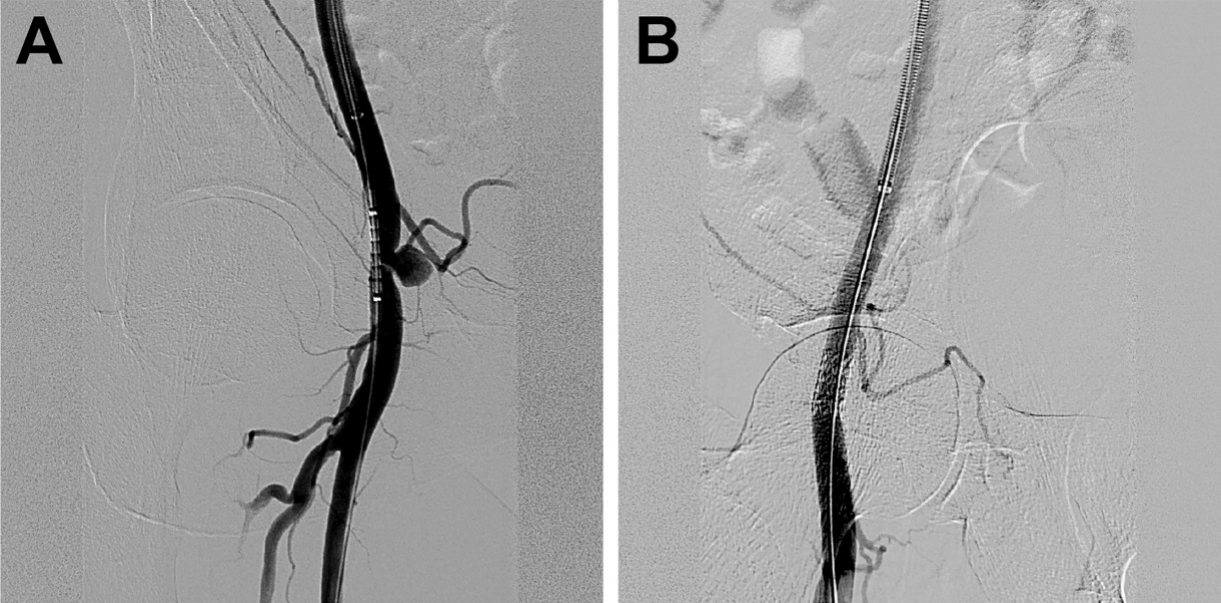
A 79-year-old male with a pulsatile mass at the puncture site and pain following percutaneous coronary intervention via femoral access. (a) Angiography using a contralateral femoral approach revealed a pseudoaneurysm. (b) A 7 x 16 mm, balloon-expanding, covered stent (LIFESTREAM; C. R. Bard, USA) was deployed. Angiography demonstrated successful sealing of the pseudoaneurysm.

Typically, the first-line therapy for iatrogenic femoral artery PA is manual ultrasound-guided compression and/or thrombin injection, depending on the size of the PA, the characteristics of the PA neck, and the presence of a concomitant AVF. Using these two approaches, PA can be treated with an estimated success rate of 97% [11]. Furthermore, thrombin injection with simultaneous balloon occlusion across the PA entry site is another promising, minimally invasive treatment option (Fig 2) [5,12]. Two currently favoured alternatives include implantation of a stent graft and open vascular surgery. Lumsden et al. [13] and Dzijan–Horn et al. [11] reported complication rates as high as 21% and 30%, respectively, for surgical repair of iatrogenic PA. In our study, three patients with PA were treated with open surgery because of their young age and rapidly expanding PAs with wide necks (> 3 mm). Implanting stent grafts is an effective and safe treatment method for iatrogenic VCs [14,15] (Fig 3). However, to the best of our knowledge, data are lacking regarding the long-term patency of this treatment strategy. Thalhammer et al. [14] reported that stent thrombosis occurred in 4/23 patients (17%) during a one-year follow-up period. Implantation of a stent graft in a so-called ‘bending area’ (i.e., the common femoral artery [CFA]) is rather controversial given the increased incidence of stent fracture and in-stent stenosis [16]. In our study, a cohort of 12 patients with PAs was treated with stent grafts. The criteria for stenting was an infected groin with extended haematoma, patient’s choice for endovascular rather than surgical treatment, and vascular surgeon consent. During the one-year follow-up period, no intermittent claudication or disabling symptoms were reported. All patients underwent colour duplex flow imaging after three months with no signs of significant (> 50%) in-stent stenosis or stent fracture. Notably, we were able to perform a 12-month follow-up colour duplex flow imaging in 40 patients. No in-stent stenosis or fracture was detected in patients treated with stents. Conversely, in a meta-analysis of patients with peripheral artery disease, the three-year patency rates after stent implantation were only 63%–66% [17]. However, the cases with peripheral artery disease included smaller vessels (e.g., superficial femoral artery and popliteal artery), which may be associated with increased rates of in-stent stenosis in arteriosclerotic vessels. Therefore, we suggest that stent grafts be used with caution in younger patients with long life expectancy, although both methods are effective and close collaboration between cardiologists, vascular surgeons, and interventionalists is imperative for appropriate patient management. If stent implantation is required, we recommend using the shortest stent graft possible. In our cohort, the mean stent length was 28.52 ± 8.78 mm.

### Dissection or arterial thrombosis

**Fig 4:**
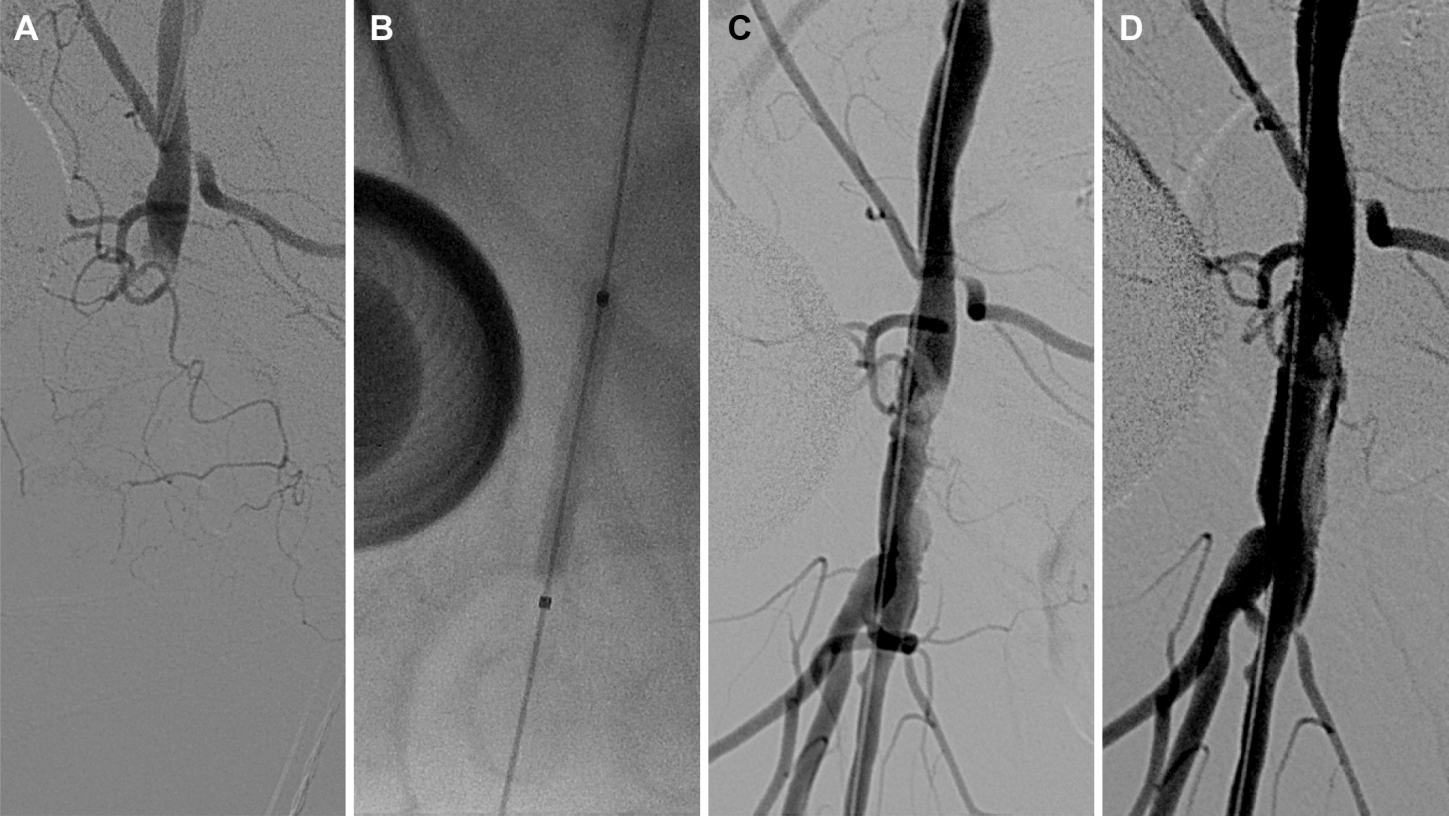
A 70-year-old woman developed acute ischemia of the right leg after percutaneous coronary intervention. (a) Total occlusion of the common femoral artery was revealed with angiography using a contralateral femoral approach. (b, c) Prolonged inflation of an angioplasty balloon was performed. (d) After completion, angiography revealed revascularization of the common femoral artery with a non-flow-limiting intimal dissection. The superficial and deep femoral arteries demonstrated patency, and stenting was not necessary.

Iatrogenic obstructive intimal dissection or arterial thrombosis after PCI can be limb-threatening, and surgical treatment involves major reconstructive procedures. Data regarding the efficiency and long-term outcomes of vascular repair of intimal dissection have rarely been reported in the literature. Endovascular strategies include prolonged inflation of a standard angioplasty balloon and/or stent grafts. Furthermore, catheter-directed thrombolysis represents another option for treatment in iatrogenic acute iliofemoral thrombotic occlusions [5]. Prolonged inflation of a balloon catheter often results in successful apposition of the intima and underlying media, especially in patients with an obstructive flap in the CFA (Fig 4). Stent grafts (spot stenting) may be necessary for persistent dissection, especially for dissections involving the external iliac artery, to sustain long-term patency [18]. We used flexible nitinol self-expanding stents (Protégé™ GPS™) in two patients with significant haemodynamic dissections in the proximal iliac artery.

### Study limitations

Our study has several limitations. Due to the retrospective single-centre nature of our study, we can demonstrate the feasibility and estimate the safety of endovascular VC treatment only in our heart centre. The low event rate does not permit direct comparisons of the likelihood of complications after the respective coronary interventional procedures or any clinical and procedural variables that can be legitimately evaluated as predictive factors. In addition, data comparing endovascular procedures with patients who underwent surgical management were limited for this study. Although we confirmed vessel and stent patency with sonography, we primarily relied on self-reported diagnoses, signs, and symptoms of claudication. Given the high morbidity of our cohort, we lost several patients during the follow-up period, resulting in an incomplete angiological follow-up, which potentially caused an underestimation of asymptomatic stent graft failure. Finally, given the limited number of patients included, the statistical power is insufficient for a thorough determination of the various potential risk factors for VC.

## Conclusions

Major femoral access-related VCs following PCI are very rare but can represent a serious event for the patients affected. Routine endovascular management in the catheterization lab seems to be a safe treatment option with a high rate of technical success, early diagnosis, and prompt treatment, especially for retroperitoneal bleeding. This technique minimises the need for surgery in the vast majority of VCs, without related risks and is associated with excellent clinical outcomes after one-year follow-up. Endovascular treatment is associated with significantly fewer red blood cell concentrates per patient and fewer hospitalisation days than surgical treatment.

We suggest storing a bailout set, consisting of catheter balloons, covered and nitinol stents of different sizes and crossover, and long sheaths for crossover and transbrachial access, in the catheterization lab for major VC cases. A basic bailout set is proposed in Table 3, which is also feasible to be provided by smaller PCI centres. An interventional cardiologist should be able to predict, prevent and, when necessary, treat iliofemoral access site complications when they do occur in bailout situations. Additional randomised controlled trials are necessary to evaluate and compare endovascular treatment with open surgical repair in bailout situations and in patients with high surgical risks.

**Table 3:** Basic bail-out set for treating iliofemoral access site complications.

Bail-out set (basic)		
**glidewire**	glidewire hydrophilic coated	(0.035” and 0.018”) 150 and 260 cm long, stiff
**sheath**	crossover sheath	7 and 8 Fr (45 cm)
**balloon**	plain angioplasty balloon	7.0–12.0 mm diameter 40 mm length
**stent (covered)**	Balloon-expandable/self-expandable	7.0–12.0 mm diameter 20–40 mm length
